# Diseases of the Stomach

**Published:** 1900-07

**Authors:** Edgar J. Spratling

**Affiliations:** Member Georgia State Medical Association, National Society for Study of Epilepsy, etc., etc.


### DISEASES OF THE STOMACH.*

By EDGAR J. SPRATLING, B.Sc., M.D.,

Member Georgia State Medical Association, National Society for Study of Epilepsy, etc., etc.

Every living organism possesses a stomach or its equivalent. From the micrococcus to man, the one common pedestal upon which all meet is appetite, catered to by a more or less well arranged stomach. Neither brain nor spinal cord, heart nor lungs, are necessary to life, but a food receptacle is a requisite. Whether it be a mere absorbent surface or an excavation in the cell-wall of the

*Read before the Georgia State Medical Association, April 19th, 1900.

organism, or an invagination by an inversion of the body itself, or the finely-adjusted, very troublesome organ of man, it serves the same purpose—the channel through which nutrition is introduced into the animal economy; and it being the prime organ by right of inception and of use, it is, as well, the one first and most prone to become deranged. Nearly all physical ailments have some relation to nutrition. Give any man a strong, healthy stomach, perfect digestion, easy assimilation, with wealth or poverty, liberty or imprisonment, and happiness in some degree will be his. Give him a stomach foul, heavy, repulsive in odor, refusing food, and unhappiness comes to him, with malevolent thoughts toward his fellow men.

A patient presents himself complaining of a vague, indefinable feeling of malaise without being exactly able to locate his trouble, yet knowing there is something wrong. He may have no appetite or he may eat too much, may be able to do his ordinary work with only slight extra effort, yet we have here cause to suspect a nutritional trouble, the very vagueness of his complaint making us expect it. We then give an examination to verify our suspicion, ask as to appetite, taste, abnormal sensations, itching or burning of the skin, dizziness, if there is belching or regurgitation of the food, if there is ever nausea or vomiting, if the bowels are regular, too frequent or infrequent. Then, if possible or convenient, we pass to inspection, if there is bulging or retraction of the stomach, if the abdominal wall seems to be girdled as in an effort to support it. This inspection is not complete until the teeth, the tongue, fauces, uvula and tonsils are seen; the nose must also have our attention.

It does not seem amiss here to refer to the condition of mouth and nose having a direct bearing upon digestion. A mouth deficient in secretion will be unable to handle the food properly, and if the salivary element is wanting, one powerful aid to digestion is needed. Should the teeth be defective in quality or deficient in number, the food will not reach the stomach in proper shape. Enlarged or painful tonsils or inflamed fauces will often prevent the patient taking a sufficiency of food to insure health. And an elongated uvula, especially if it trails the back of the tongue, will create nausea and disgust that kill the desire for food. Diseased nostrils will not only abolish the appetite by their offensive secretion in the person possessing them, but be a very sure damper upon the gastronomic enjoyment of those near. Nasal so frequently precedes gastric catarrh.

After this inspection we pass to palpation. We go over stomach and intestinal track, noting the sensitive areas, tumefactions, the throb of the aorta, if the stomach extends beyond its normal limits, if the intestines, especially the colon, present gaseous peculiarities. Should we be unable to outline the stomach, it being desirable, we introduce half drachm or so each of soda bicarbonate and tartaric acid, separately dissolved in water, which gives us dilatation, when the stomach walls may be easily outlined. The same procedure may be used in the colon, care being taken in each case that we are not dealing with organic lesions for fear of rents being made.

Auscultation often aids us. We can hear fluid passing through the esophagus, and get the splashing sound of the stomach when partly filled with water. Sometimes, but not often, the so-called respiratory sound may be noted, which is produced by the stomach gliding against the abdominal walls. Sizzling sounds are frequently heard in the fermentative dyspepsias. The interior of the stomach may be inspected directly by the gastroscope, which is an adaptation of the long-used cystoscope. Practically, this is without value. Transillumination is a little more to be recommended, as we have here only to let the patient swallow an electric light and note the translucency of the walls. Our most reliable aid in diagnosing the stomach is the test-tube. And here our first step is the administration of a test-meal. Let the patient eat freely of soup, meat, potatoes and bread and a cup of tea. In the first instance the food should be allowed to remain in the stomach at least four hours before being withdrawn for examination; in the second, one hour is a sufficient length of time. Some authors prefer a pint of milk and a roll, the examination being delayed about two hours after its ingestion. Every test-meal should be administered after the patient has fasted at least seven hours. If it is possible to voluntarily expel the contents it is desirable to do so. In that way we get less newly exuded juices, which are rapidly thrown out under the irritation of the stomach-tube or of any emetic.

After we have obtained the contents we note whether it is a well-mixed chyme or whether there are still large food particles suspended in it, and in case of test dinner note whether we find particles of bread, potatoes or meat. Then we use the microscope, noting whether there are oil globules, and their condition; if there be meat, whether the muscle fibers have been attacked or whether the sarcolemma is yet intact; if there are starchy particles, whether granules are entire or ruptured. Next we take the reaction. If acid, we try for hydrochloric acid by the following test: Resorcin five, sugar three, and alcohol to one hundred; make a mixture, and allow a drop of this to mix with a drop of stomach contents. On heating this will develop a cherry red. For lactic acid we test with a two per cent. solution of carbolic acid in water having a drop of the sesquichloride of iron in it; to this in test-tube we add the filtrate, obtaining a canary-yellow. This has the advantage of finding also the fatty acids as well as the inorganic, the first giving an ashy-gray color, the second decolorizing the solution entirely. In this test for lactic acid we are sometimes confused by the phosphates, which may be first removed by treating with ether. Propeptone is found by a solution of sodium chloride, which gives a white precipitate. The peptone may be made out by addition of sodium hydrate followed by a few drops of sulphate of copper solution, getting a purplish color. A free pepsin may be found by placing a thin disk of egg albumen in a test-tube of stomach contents. It ought to be entirely dissolved in five hours. Rennet may be noted by mixing with milk and keeping warm, getting curds. The starches are easily discovered by a drop of Lugol's solution.

The stomach-tube, even for experimental purposes, should be used with caution where we have reason to suspect the presence of organic disease, especially of malignant type. This applies as well to the so-called stomach-bucket as to the tube, even the introduction of the sponge rarely being dangerous, in that it may bring on contractions that result seriously. No instrument should be introduced into a stomach until a reasonable time has elapsed since any vomiting of blood. The instruments for determining motility are without practical value, as we soon learn from experience whether a stomach be sufficiently active to make its contents pass on into the intestines.

Of all subjects that arise in the practice of medicine none gives the physician more uneasiness, more worry and frequent disappointments, than diet. The Latin proverb, "So many men, so many minds," could be equally well said, So many men, so many stomachs, for, as every man's appetite is a law unto itself, so is every stomach governed by its own peculiarities. The mental impressions of the patient, the season of the year, and innumerable and uncontrollable factors must enter into our consideration if we wish to have the patient pleased with our handling. One patient comes to us unable to eat sweets, another declares that meat of every description is abhorrent, while the third regrets that he cannot touch vegetables without distress, fats are both repugnant and harmful to the fourth, and even milk, our sheet-anchor, is refused by a considerable contingent. These facts, taken in connection with the iron-clad necessity of replacing as much nutrition as there is used up and wasted by the animal economy, force us to our utmost efforts catering to the whims of each stomach.

Our whole aim in acute gastritis is to feed the body while letting the stomach rest. We may accomplish this by giving predigested, easily-absorbed foods by mouth, or even at times enemata, or we can for a short time depend upon the reserve force to sustain the individual. This should be avoided wherever possible, as the tissue waste will have to be made up later.

About the blandest diet that can be depended upon for nutritive purposes would consist of variation of bouillon, meat extract and solutions, milk, raw eggs, egg water, crackers, boiled brains and fowls and meat gruels. No patient should be kept on a diet too bland, as such management would inevitably work injury by getting the organ out of the habit of exercising its normal functions.

The most frequent disease of the stomach upon which the physician is called to exercise his care is acute catarrh, or simple gastritis. Here we would only give the organ rest and conserve the physical energies, and recovery is quickly had. When catarrh has become chronic, lavage and spraying the stomach walls is our surest remedy This condition is easily diagnosed. A patient comes complaining of weighty fullness, decidedly abnormal appetite, furred tongue, sweetish breath, irregular dreamful sleep, and we have sufficient evidence for the use of the tube to verify our diagnosis of gastric catarrh—always barring, of course, cases in which we are led further to suspect an actual organic lesion other than ulcer.

Ulcer ranks next in importance and frequency. Here our diagnosis is equally easy. We get all the symptoms of gastric catarrh, and in addition local tenderness and possibly hematemesis. Perhaps the majority of authors condemn the use of the tube for diagnostic purposes. In case of suspected ulcers this objection is without foundation, as with a soft tube and care in introducing there is no reason whatever for doing an injury, except it is done by the unusual contractions of the stomach. This can be avoided by the use of cocain or morphine. Here the gastric juice is so powerfully acid that the tube with its local applications is more demanded actually than in any other stomach trouble. It is manifestly out of the question to treat an ulcer on the wall of the stomach by medication to its surface, because it is so protected by thickened secretions that no reasonable amount of medicine ingested can touch or can benfit it. With this one single exception, tonics and hematics generally are indicated as aiding the reparative power of the system. The futility of medication here is further emphasized by the fact that the majority of ulcers are situated on the back and upper surfaces of the stomach, while the medicine seeks the pendant portions. The importance of diagnosis and treatment of the ulcer by the general practitioner is seen when we say that one in five of adults at necropsy is shown to have suffered from it.

Epithelioma, while not so frequent an affection, yet one or several cases usually come into the life of every practitioner. The ability to diagnose and prognose is greatly to be desired. Without the aid of the microscope in the early stages it is practically impossible to distinguish between epithelioma and ulcer. Carcinoma ventriculi is shown by statistics to be increasing more rapidly than any other fatal disease that affects humanity. It is essentially a disease of full-grown active manhood or womanhood, coming when the individual is worth most to himself, more to his family and to society. This is why its diagnosis is most important.

Hyperchlorhydria ranks first in importance and in frequency of functional disturbances of the stomach. Its diagnosis is most easy, but the prognosis as to the continuance of the trouble is very uncertain, though we are able always to assure the patient that it is not to be fatal.

Excessive secretions of all sorts frequently come to our attention. The opposite condition of achylia gastrica is rarely met with, though when it does occur it is perhaps the most disheartening, most annoying and discouraging that can affect a patient. By this term we mean the normal secretions of the stomach are absent. It usually is produced by an actual atrophy of the gastric glands· which defect is insuperable, leaving us at the mercy of predigested or otherwise easily absorbed foods.

Of the gastric neuroses we need only give a mere mention of bulimia, which is the so-called morbid appetite, the patient never being satisfied and always hungry. This is often due to foreign substances, either living parasites or some solid body swallowed. Our only resource is to find and remove the cause.

The motor neuroses do not properly come under the head of the diseases of the stomach, but under the care of the neurologist.

The relations between the diseases of the stomach and those of the other organs of the body is very close. We have all been taught by many a sad experience to look with suspicion upon an indigestion that has arisen without any apparent indiscretion in diet and perhaps accompanied by just the slightest hacking cough. The kidney makes its own impression upon the digestion. Almost every heart lesion gives us a thickened, heavy coating to the stomach.

On the other hand, the stomach is the gateway leading to innumerable disorders. It does this by both direct impressions and by lowering the body nutrition. I do not venture contradiction when I say that ninety cases of every hundred in epilepsy come reflexly in the beginning from the stomach. Those of us who have dealt with the defective of every class—with the lunatic, the epileptic, the imbecile, the idiot, and even the criminal—have found by experience that their emotions are swayed by their actions determined largely by the digestion.

